# Standardization of dot-enzyme-linked immmunosorbent assay for the diagnosis of bovine visceral schistosomiasis

**DOI:** 10.14202/vetworld.2017.536-541

**Published:** 2017-05-21

**Authors:** Kommu Sudhakar, G. S. Sreenivasa Murthy, Gaddam Rajeshwari

**Affiliations:** 1 Department of Veterinary Parasitology, College of Veterinary Science, P V Narsimha Rao Telangana Veterinary University, Rajendranagar, Hyderabad, Telangana, India; 2 Department of Veterinary Parasitology, Teaching Veterinary Clinical Complex, College of Veterinary Science, P V Narsimha Rao Telangana Veterinary University, Rajendranagar, Hyderabad, Telangana, India

**Keywords:** dot-enzyme-linked immmunosorbent assay, excretory-secretory antigen, *Schistosoma spindale*, whole worm antigen

## Abstract

**Aim:**

Bovine visceral schistosomiasis has been reported as an important disease entity as it affects animal health, productivity, causes economic losses due to liver condemnation, and produces a high morbidity. This study was conducted to standardize an easy, reliable dot-enzyme-linked immmunosorbent assay (ELISA) for the diagnosis of visceral schistosomiasis caused by Schistosoma spindale and to know the prevalence rate in and around Hyderabad.

**Materials and Methods:**

A dot-ELISA was standardized in the laboratory using whole worm antigen (WWA) and excretory-secretory antigen (ESA) of S. spindale. The standardized test was used for the diagnosis of bovine visceral schistosomiasis at field level. The sensitivity and specificity of the test was compared with counter current immunoelectrophoresis. In total, 288 sera (125 cattle and 163 buffalo) were screened by dot-ELISA.

**Results:**

The dot-ELISA detected 32.63% of infection (94/288) using WWA and 40.62% of infection (117/288) using ESA. In cattle, the prevalence rate was 32.80% (41/125) using WWA and 40.80% (51/125) of infection. Similarly, in buffaloes, the prevalence rate was 32.51% (53/163) using WWA and 40.49% (66/163) of infection using ESA. The overall sensitivity of dot-ELISA was 76.74% and 80.48% with WWA and ESA, respectively, and specificity was 73.3% and 78.57% in WWA and ESA, respectively.

**Conclusion:**

As ante-mortem diagnosis of visceral schistosomiasis is difficult in subclinical conditions, dot-ELISA can be used as a reliable immunodiagnostic test for diagnosis at field level.

## Introduction

Schistosomiasis has been recognized as one of the major parasitic diseases of livestock and human beings. Bovine visceral schistosomiasis in Indian subcontinent is primarily caused by two *Schistosoma* species such as *Schistosoma spindale* and *Schistosoma indicum*. High rates of prevalence of subclinical infections cause significant losses due to long-term effects on animal growth, productivity, reduced conception and pregnancy rates [1], and increased susceptibility to other parasitic or bacterial diseases [1,2]. Schistosomiasis also causes loss due to liver condemnation in animal species [3]. All fecal examination methods for diagnosis are found to be less sensitive [4]. *S. spindale* infection is generally underdiagnosed, as the eggs in fecal samples of affected animals are usually masked by high mucus content and eggs in feces also hatch immediately on contact with water. Previous studies on the incidence and prevalence of schistosomiasis in India were based solely on nasal scrapings, fecal examination, and nasal and mesenteric cuttings in cattle [5]. A coprological survey in water buffaloes of Kurigram district of Bangladesh indicated 1.27% *S. indicum* and 0.85% *S. spindale* infection [6]. This trematode was more likely to co-occur with other gastrointestinal parasites (i.e., *Dicrocoelium* spp., *Paramphistomum* spp., *Strongyle* spp., *Eimeria* spp., and *Entamoeba* spp.), and statistical analysis revealed that female cattle are less likely to get *S. spindale* infection as compared to male cattle, and cattle weighing lower than 200 kg were significantly at higher risk than those higher than 200 kg in Malaysia [7].

The antigens from schistosome eggs, cercariae, and schistosomula have been isolated and found useful for various purposes. The secretory-excretory products, tegument, and gut proteins of adult schistosomes are the source of potential immunogens. Very less attempts were made toward the identification, characterization, and use of antigens of *S. spindale* toward these objectives. The immunodiagnosis can be considered as an essential mean for the confirmatory diagnosis.

Hence, the present study was conducted to standardize a serological test (dot-enzyme-linked immmunosorbent assay [ELISA]) for the diagnosis of visceral schistosomiasis and to evaluate it for the diagnosis of the disease in field levels.

## Materials and Methods

### Ethical approval

Two rabbits were used for raising hyperimmune sera (HIS) against whole worm antigen (WWA) and ES antigen (ESA) of *S. spindale* (permission was accorded by Ethical Committee bearing Ref: 698/CPCSEA dated October 01, 2002 F.No. 25/60/2010-AWD/Veterinary College/Hyderabad).

### Collection of adult schistosomes

The mesenteric veins of the freshly collected bovine intestines from slaughter house were examined against sunlight, and the adult schistosomes were collected carefully in sterile phosphate-buffered saline (PBS). After three washings in sterile PBS, the parasites were segregated species wise based on their morphological characters under light microscopy. *S. spindale* male worms were identified by their smooth cuticle with 3-5 number of testes and female worms lodged in gynecophoric canal of male worms containing spindle-shaped eggs in the uterus with terminal spine at one end. *S. indicum* worms can be separated based on rough cuticle (tuberculated/spiny) with 5-7 testes and the female worms containing oval-shaped eggs with terminal spine at one end. After species separation, *S. spindale* worms were used for further studies.

Around 500 freshly collected live flukes were washed thoroughly in normal saline and transferred to 2 ml of 0.01 M PBS (pH 7.4) containing ampicillin (40 mg), amikacin (40 mg), sodium azide (0.1%), protease inhibitor, and phenylmethylsulfonyl fluoride (PMSF 1 mM). The worms were incubated at 37°C in an incubator with 5% carbon dioxide for overnight. On next day morning, the incubated fluid containing excretory-secretory (ES) products was collected and centrifuged at 13,000 rpm at 4°C for 30 min. The supernatant was collected and stored at −20°C.

Similarly, the WWA were prepared by taking around 500 worms of *S. spindale* after washing thrice in PBS (pH 7.4) and triturated with 5 ml of PBS (pH 7.4) using glass tissue homogenizer (Potter Elvehjem glass Teflon) for 10 min at 4°C. The contents were sonicated at 20 kHz for 10 cycles for 90 s each with an interval of 1 min in between. The contents were centrifuged at 9500 ×*g* for 15 min at 4°C in a high-speed centrifuge (C30 Remi, India). The supernatant was collected and preserved by adding one drop of 1% sodium azide and 1 mM of PMSF at −20°C, after making the solution into several aliquots.

The protein concentration of antigens was estimated as per the method of Lowry *et al*. (1951) using protein estimation kit obtained from Genei, Bengaluru.

### Protocol for raising of hyper immune sera

WWA (0.25 ml) containing 3.88 mg/ml of protein along with equal volume of Freund’s complete adjuvant (FCA) was injected subcutaneously into one rabbit. Similarly, 0.3 ml of ESA (2.01 mg/ml of protein) along with 0.3 ml of FCA was injected subcutaneously to another rabbit. Four booster doses containing either WWA (0.25 ml) or ESA (0.3 ml) along with equal volume of Freund’s incomplete adjuvant were administered subcutaneously to the respective rabbits at weekly intervals. The animals were bled by the puncture of marginal ear vein and checked for antibody (Ab) response by agar gel precipitation test before administering each booster injection. Blood was collected by cardiac puncture on the 10^th^ day of last booster injection. Serum was separated under sterile conditions, and complement inactivation was done by keeping in water bath at 56°C for 30 min. Sera were tested for the presence of antibodies using corresponding antigen with double immunodiffusion (DID) and counter current immunoelectrophoresis (CCIEP) techniques. The sera containing antibodies for respective antigens were made into aliquots and stored at −20°C till further use.

The immunoprecipitation reactions between antigen (WWA/ESA) with respective hyperimmune sera developed in rabbits were detected by DID and CCIEP.

### Standardization of dot-ELISA using HIS

Dot-ELISA was standardized by charging 1.5 µl of different dilutions of the antigens (3.88, 1.94, 0.97, and 0.485 mg/ml of WWA and 2.01, 1, 0.5, and 0.25 mg/ml of ESA) on to nitrocellulose (NC) membrane of 1 cm^2^, which was previously washed in autoclaved double-distilled water and dried at room temperature. The strips were kept in the incubator at 37°C for 1 h. The serum samples (HIS and negative controls) at different dilutions (1:50, 1:100, 1:200, and 1:400) with 0.05% Tween 20 in PBS (PBST) were allowed to react with the antigen-loaded strip for 1 h at 37°C followed by washing of NC strips for 6 times in PBST (pH 7.4). The NC membranes were treated with goat anti-rabbit immunoglobulin G (IgG) horseradish peroxidase (HRP) conjugate at different dilutions (1:1000, 1:1500, 1:2000, 1:2500, and 1:5000) with PBST followed by washing 4 times each for 10 min. Subsequently, the Ab and conjugate-treated antigen-coated NC membrane strips were finally treated with the chromogen substrate di-aminobenzidine (DAB - 0.5 mg/ml, cobalt chloride - 0.2 mg/ml, Tris buffer - 0.05 M, sodium chloride - 0.15 M, and 3% hydrogen peroxide – 15 µl) in dark for 2-3 min. The dark brown spot appeared on the NC strip indicated positive reaction. The reaction was stopped with double-distilled water. Irrespective of the intensity of the color, the reaction was considered as positive and was documented by digital zoom camera (Nikon COOL PIX AW 120).

The lowest possible concentration of either WWA or ESA giving positive reaction with highest possible dilution of respective HIS and goat anti-IgG HRP conjugate in the form of brown spot on NC membrane, irrespective of color intensity, was considered as cutoff value and the same was taken as standard dilution for the screening of bovine serum samples from the field.

### CCIEP

CCIEP was also done to compare the sensitivity and specificity of the standardized dot-ELISA. Agarose was prepared in Tris borate buffer (pH 8.2). 1 g of agarose special, low EEO (HIMEDIA), was boiled in 100 ml of Tris borate buffer. 5 ml of molten gel was poured on to glass slides and allowed to solidify. Two wells of 4 mm diameter were cut with a well distance of 5 mm for trials with corresponding hyperimmune sera. The slides were connected by a filter paper wick dipped in buffer. A current of 50 mA per slide was applied and results were recorded after 90 min.

The sensitivity and specificity of dot-ELISA was measured by standardized dot-ELISA procedure described as earlier.

To evaluate the sensitivity and specificity of CCIEP, two rows of wells, each row having seven wells (each well having 4 mm diameter and a distance of 5 mm in between), were punched on agar slide. Wells were cut and the bases were sealed with molten agar. The well to be located on the cathode side was filled with antigens of *S. spindale* and other (anodic well) with corresponding hyperimmune serum for evaluation of the test and later with sera from known positive or negative animal. Electrophoresis was carried out in suitable trough containing electrophoresis buffer. Both ends of the slides were connected with a filter paper wick dipped in buffer. A current of 50 mA per slide was applied for 90 min. The slides were washed in normal saline and stained with Coomassie brilliant blue.

The sensitivity of dot-ELISA and CCIEP was measured by screening 33 known positive sera collected from cattle which were later confirmed by finding schistosomes in the mesentery on post-slaughter examination.

The specificity of laboratory dot-ELISA and CCIEP was measured by screening the sera of cattle reared in confined area and was found negative for the clinical symptoms of schistosomiasis or any other helminth ova in the feces.

The laboratory-standardized dot-ELISA was tested for its efficacy in the diagnosis of bovine visceral schistosomiasis and further evaluation of dot-ELISA at field level.

Around 288 bovine sera were collected from different parts in and around Hyderabad, Telangana state (erstwhile Andhra Pradesh) and subjected to standardized dot-ELISA with different antigens.

## Results

**Figure-1:**
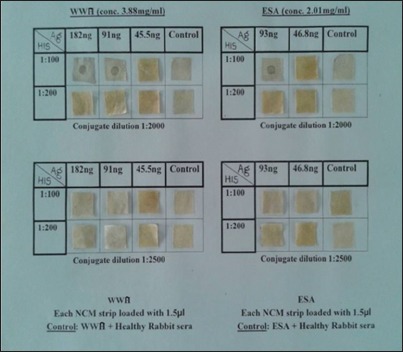
Optimum concentration of antigen (whole worm antigen/excretory-secretory antigen), hyperimmune sera, and conjugate (goat anti-rabbit immunoglobulin G horseradish peroxidase) showing a brown dot.

The dot-ELISA was standardized at an antigen concentration of 91 ng for WWA and 93 ng for ESA which gave brown color dot with the chromogen substrate DAB after reacting with the serum and conjugate at 1:100 and 1:2000 dilutions, respectively (Figure-1).

The overall sensitivity of dot-ELISA was determined as 76.74% and 80.48% with WWA and ESA, respectively, and specificity was 73.33% and 78.57% in WWA and ESA, respectively (Tables-1 and 2). A total of 288 bovine sera (125 cattle and 163 buffalo) were examined using the standardized dot-ELISA test. Out of the 125 sera samples screened in cattle, 41 were positive indicating 32.80% infection with WWA and 51 were positive indicating 40.80% infection with ESA. Whereas in the screening of 163 sera samples in buffalo, 53 were positive indicating 32.51% infection with WWA and 66 were positive indicating 40.49% infection with ESA. The overall percentage infection of schistosomiasis in bovines was recorded as 32.63% (94/288) using WWA and 40.62% (117/288) using ESA (Table-3).

**Table-1:** Sensitivity studies on dot-ELISA in *S. spindale* infection (WWA and ESA).

Antigen	Number of samples (known positive)	Dot-ELISA (positive)	Dot-ELISA negative (false negative)	Sensitivity percentage
WWA	33	23^a^	10	76.74
ESA	33	25^a^	8	80.48

Sensitivity: True positive/True positive+False negative×100. The values superscripted with similar alphabets are not significantly (p≥0) different. WWA=Whole worm antigen, ESA=Excretory-secretory antigen, ELISA=Enzyme-linked immmunosorbent assay,*S. spindale=Schistosoma spindale*

**Table-2:** Specificity studies on dot-ELISA in *S. spindale* infection (WWA and ESA).

Antigen type	Number of samples (known negative)	Dot-ELISA (negative)	Dot-ELISA positive (false positive)	Specificity percentage
WWA	11	7	4	73.33
ESA	11	8	3	78.57

Specificity: True negatives/True negatives+False positives×100. The values superscripted with similar alphabets are not significantly (p≥0) different. WWA=Whole worm antigen, ESA=Excretory-secretory antigen, ELISA=Enzyme-linked immmunosorbent assay,*S. spindale=Schistosoma spindale*

**Table-3:** Detection of *S. spindale* infection by dot-ELISA using WWA and ESA.

Species	Number of sera screened	Dot-ELISA using WWA				Dot-ELISA using ESA			
	
		Positive	Negative	Percent- positive infection	Percent- negative infection	Positive	Negative	Percent- positive infection	Percent-negative infection
Cattle	125	41^by^	84	32.80	62.20	51^ay^	74	40.80	59.20
Buffalo	163	53^bx^	110	32.51	67.49	66^ax^	97	40.49	59.51
Total	288	94	194	32.63	67.37	117	171	40.62	59.38

ab The positive values bearing different superscriptions between the species differ significantly (p≤0.05).

xy The positive values bearing different superscriptions between the tests differ significantly (p≤0.05). WWA=Whole worm antigen, ESA=Excretory-secretory antigen, ELISA=Enzyme-linked immmunosorbent assay,*S. spindale=Schistosoma spindale*

## Discussion

Schistosomiasis has been recognized as one of the major parasitic diseases of livestock and human beings. *S. spindale* is the major cause of visceral schistosomiasis among bovines [8]. Routine diagnostic methods have poor sensitivity and thus underestimate the actual prevalence which ultimately interferes with the control strategies [9]. The prevalence of schistosomiasis was very high based on mesenteric worm technique when compared to the existing reports based on fecal egg detection techniques [9]. This study emphasizes the need for developing alternate field-level diagnostic tests for schistosomiasis because the routine method of diagnosis generally underdiagnoses many infections at field level.

Dot-ELISA, a modification of the standard ELISA assay, offers a practical tool for field studies. Moreover, the control of a disease is possible only based on the sensitivity and specificity of the diagnostic method. Dot-ELISA technique was first applied for detecting the anti-leishmania antibodies in human beings.

Different immunological techniques, namely, latex agglutination test, sandwich ELISA, and dot-ELISA were evaluated for the diagnosis of *Schistosoma haematobium* infection in Egypt using purified soluble egg antigen, and the tests were proved to be valuable and can be used as an easy, fast, and accurate diagnostic technique [10].

In the present investigation, 91 ng of WWA and 93 ng of ESA of *S. spindale* dotted on NCM could give a positive brown-colored dot with 1:100 dilution of HIS when coupled with goat anti-rabbit IgG HRP followed by reaction with DAB substrate.

Various authors have tried to develop different kinds of ELISA tests including dot-ELISA which required antigens in nanogram quantities and the present findings were similar to them [8,11-15].

The dot-ELISA using WWA could detect 23 positive cases out of 33 known positive bovine sera, indicating an overall sensitivity of 76.74% whereas the same test with ESA detected 25 positive cases among 33 known positive bovine sera showing an overall sensitivity of 80.48%. Similarly, dot-ELISA using WWA gave 4 positive reactions with 11 known negative sera showing 73.33% of specificity. Whereas ESA reacted positively with 3 out of 11 known bovine sera negative for schistosomiasis indicating 78.57% of specificity. Our results were in correlation with the findings of earlier workers [8,13,15]. In the present study, schistosomiasis was detected in 32.63% and 40.62% of bovines in Telangana, using WWA and ESA in dot-ELISA, respectively. These results are comparable with that of Lakshmanan *et al*. [8] as 32% of bovines in Kerala using ESA in dot-ELISA. The results are almost in agreement with that of Lakshmanan *et al*. [8], but they have not categorized as cattle or buffaloes like in the present findings.

Adult worm homogenates of *S. indicum* and *S. spindale* were prepared and subjected to sodium dodecyl sulfate polyacrylamide gel electrophoresis to know the polypeptide profiles of the prepared antigen. Finally, four immunodominant proteins (45, 40, 28, and 15 kDa) were obtained and no significant variation was found between the immunodominant proteins *S. indicum* and *S. spindale*. As mixed infection is common in bovines, in this study, the mixed worm antigen was used for the detection of humoral immune status of infected cattle, sheep, and goats [12].

Cattle sera showed 32.8% (41/125) infection with WWA, 40.80% (51/125) infection with ESA, whereas buffalo sera showed 32.51% (53/163) infection with WWA and 40.49% (66/163) infection with ESA. Use of WWA in dot-ELISA detected anti-schistosomal antibodies in cattle indicating 90.93% (291/320) [11]. The results of the present findings in cattle are not corroborated with that of Sumanth *et al*. [11] who advocated positivity in latter cases either due to light infection or their previous exposure to infection, most probably this was due to non-specificity of the reaction.

In this study, buffaloes showed 32.51% and 40.49% infection using WWA and ESA, respectively. Results cannot be compared, due to the paucity of literature, with various authors as they reported either in cattle or in general bovines, but the present study shows a significant infection rate of schistosomiasis in cattle and buffaloes separately in Telangana state.

As a whole, the dot-ELISA with ESA was found to be more sensitive (80.48%) and specific (78.57%) than WWA in detecting antibodies to *S. spindale* infection in bovines. The superiority of ESA in serodiagnosis has been observed by several workers for detecting other trematode infections, namely, *Clonorchis sinensis, Paragonimus* spp., *Fasciola hepatica* [16], *Opisthorchis felineus* [17], and *Paramphistomum cervi* [18]. This ESA could be useful for rapid screening of a large number of field samples, with wide applicability.

Even though some differences have been identified regarding the sensitivity and specificity of the dot-ELISA in its different applications [19,20], the test was proved to be highly stable, does not require specialized tools to analyze the results, cost-effective, and can be simultaneously applied on large-scale screening of samples according to Chieffi *et al*. [21].

## Conclusion

It is concluded that *S. spindale* antigens were useful for serodiagnosis of visceral schistosomiasis in bovines by dot-ELISA. Dot-ELISA using ESA could also be considered for ante-mortem diagnosis of schistosomal infection with a high degree of reliability under field conditions. This technique could form the basis for evolving a uniform system of seromonitoring of *S. spindale* in local bovine population, in turn enabling implementation of a suitable disease control strategy in the country.

## Authors’ Contributions

GSSM and KS have designed the concept and supervised the plan of work and also have prepared the manuscript. GR helped in collecting intestines from slaughter house and provided technical, as well as material support. GSSM analyzed and interpreted the data. All authors read and approved the final manuscript.
